# Climbing the Ladder of Fame

**Published:** 1881-05

**Authors:** 


### CLIMBING THE LADDER OF FAME.

### THE PARENTAGE AND SUBSEQUENT LIFE OF OUR PRESIDENTS.

[Extract from Letter in Philadelphia Times ]

WASHINGTON belonged to the highest classes and was known as a "Virginia gentleman." His family belonged to the aristocracy and was connected with the English gentry. He began life as a surveyor, but his social position was increased and he obtained wealth by marrying the Widow Custis. A story is told of his later years that a poor Virginian of quality, whom he had reproved, retorted sharply, "I should like to know, George Washington, what you would have been if you hadn't married the Widow Custis." Washington smiled, because the man was poor and unfortunate, but he rarely allowed such liberties.

John Adams was the son of a farmer and shoemaker, although his family had been settled in Massachusetts for seven generations. The Adamses are one of a few American families that can be called historical. They are known in politics, law, literature and statesmanship. They are also known for their wealth. No family can compare with them in the number of offices they have filled and the respect in which they are entertained. In ancient Rome they would have been called a consular family. They are the Adamses of Quincy, and in France Charles Francis Adams would be called M. de Quincy. Quincy is an old French, or rather a Norman name, and appears in the roll of Battle Abbey. It has only a territorial relation to the Adamses. The name belongs properly to another great American family, descended from an ancestor who was in the battle of Hastings, A. D. 1066. John Adams was a lawyer by profession.

Thomas Jefferson was also a lawyer. His family held a good, but not a high, social rank in Virginia. He made for himself a great name for all time as a legislator, an author, a diplomatist and a political leader, but his greatest achievement was the writing of the Declaration of Independence. He came into public life through the struggle between the Colonies and England.

James Madison came from a wealthy family of Virginia planters. He was a lawyer, but never practiced much at the bar, having entered public life very early. He was a great student.

James Monroe was also the son of a Virginia planter, and a lawyer, but, like Madison, he went early into public life. He served honorably in the army and in many civil offices. He had a wide experience in public affairs and a thorough training in statesmanship.

John Quincy Adams was the oldest son of John Adams, and a lawyer; but his life was devoted to public service, with a few interludes given to literary pursuits. He was educated for statesmanship by his parents, and had a larger and more varied experirience in public life than any other President.

Andrew Jackson was a poor boy, whose early life was full of hardships. He came from a poor Southern family, and at manhood began the practice of law. His pursuits were various. He was a politician, and served in both houses of Congress. He was also a merchant, a judge, a planter and a soldier.

Martin Van Buren's father was too poor to give him an education, and whatever he was he owed to his own exertions. He struggled along, as many a poor boy is doing to-day, and eventually obtained a fair education and was admitted to the bar, where he achieved a high position.

Wm. Henry Harrison's father was by no means rich, but a Virginia planter in comfortable circumstances. He was a signer of the Declaration of Independence. The son entered the army early and his accounts as Commissary may now be seen in the Treasury Department, and are fine specimens of clerical skill. He occupied several trusts in civil life.

John Tyler was the son of an eminent Virginian, who occupied many high public offices. Tyler himself was admitted to the bar when but 19 years old. He was for many years in the State Legislature and was Speaker of the Assembly. He was also a member of Congress.

James K. Polk was the son of a farmer in moderate circumstances, and was a clerk in a store. He subsequently studied law and was a member of the Tennessee Legislature for five terms. He was fourteen years in Congress, and, refusing a re-election, was chosen Governor of his State.

Zachary Taylor was the son of poor parents. His father was Colonel Richard Taylor, who won distinction in the Revolution.

He emigrated to Kentucky soon after his son's birth. The son's life, after leaving the plantation, was spent in the army. He was on duty in Louisiana when elected President, and he remained at his post until he came to Washington to be inaugurated.

Millard Fillmore was of the humblest origin, and his early life was spent in close poverty. He served an apprenticeship of five years to the fuller's trade. He subsequently educated himself and became a lawyer.

Franklin Pierce was the son of General Benjamin Pierce, a Revolutionary officer. He taught school and studied law, but as a lawyer he was not distinguished. He was in both houses of Congress, and when he entered that body was the youngest member of the Senate. He was a brigadier-general in the Mexican war.

James Buchanan was the son of an Irishman from the county of Donegal, who early settled in Pennsylvania. He studied law, held several offices in the State, was in both houses of Congress, and Minister to England. His public life was long and active.

Abraham Lincoln, as every one knows, was a rail splitter and flatboatman, who educated himself, became a great lawyer, and was in Congress. As a writer of the English language he had no superior.

Andrew Johnson was of the lowest origin. He learned the trade of a tailor and was so ignorant when he arrived at man's estate that he could neither read nor write. His wife taught him both after he was married. He was in the State Legislature and in both Houses of Congress.

Ulysses S. Grant was the son of an obscure man in humble circumstances. He graduated from West Point, but resigned from the Army and became very poor. He subsequently became the greatest soldier of his time.

Rutherford B. Hayes——

And now James A. Garfield may be regarded as of humble origin —a canal boatman, a school teacher, a college professor, a preacher, a lawyer, a legislator, a soldier, a lecturer, a Congressman, etc.

It will be seen from the above that more than four-fifths of the Presidents of the United States have been lawyers, and it will also be seen that nearly half of them have been of humble origin. There is a chance for the boys in Girard College yet, and they had better improve it.

---

YOUNG sportsman wants to know "What is the best kind of a dog for me to buy?" A dead one, Sporty, and have it buried the same day you buy it.—*Keokuk Gate City.*

